# Supplementary material for: A Likelihood Approach for Real-Time Calibration of Stochastic Compartmental Epidemic Models
Source: PLoS Comput Biol. 2017 Jan 17;13(1):e1005257. doi: 10.1371/journal.pcbi.1005257 (PMC5240920; doi:10.1371/journal.pcbi.1005257)
Supplement: S1 File — (TAR.GZ) [file pcbi.1005257.s014.tar.gz › HSPH_Online-SI-Revision/PF08/n10-mild/n10-mild_PF_table0.pdf]

1	R0	{1.25046, 6.80278}	{1.54679, 19.7008}	{{1.51756, 19.2569}, {1.57413, 20.}}	{23.698, 189.599}	inf	157.	815.902	{748.372, 866.258}	419.683	0.715117	newly	174.	291.23	{263., 320.}	67.3736	0.222469	Reff	1.06127	1.03976	{1.01662, 1.06223}	2.13229	ar	3516.	7248.14	{7012., 7471.}	106.147
2	R0	{1.32013, 4.45847}	{1.91015, 19.4136}	{{1.67078, 16.8918}, {2.1481, 20.}}	{44.6941, 335.433}	inf	321.	1383.83	{1007.58, 1642.45}	331.098	0.627202	newly	488.	507.88	{418., 589.}	11.3484	0.0481243	Reff	0.980989	1.19958	{1.11201, 1.2738}	22.2827	ar	4420.	8716.39	{7904., 9280.}	97.2034
3	R0	{1.31785, 5.36796}	{1.92211, 17.6423}	{{1.53548, 11.5435}, {2.19747, 20.}}	{45.852, 228.659}	inf	363.	1347.42	{790.142, 1814.43}	271.191	0.551201	newly	488.	551.09	{448., 668.}	16.834	0.0664382	Reff	1.00407	1.28798	{1.10588, 1.41694}	28.2761	ar	4942.	8498.48	{7019., 9308.}	71.9644
4	R0	{1.38801, 6.16031}	{1.68181, 19.6524}	{{1.55372, 19.0599}, {1.82853, 20.}}	{21.1672, 219.016}	inf	457.	1115.57	{866.041, 1409.31}	144.108	0.380794	newly	527.	399.76	{308., 494.}	24.425	0.127803	Reff	1.01047	1.09998	{1.04482, 1.14979}	9.18032	ar	4790.	7829.61	{7235., 8453.}	63.4574
5	R0	{1.27955, 11.57}	{1.51953, 19.5289}	{{1.5115, 19.427}, {1.53027, 19.5901}}	{18.755, 68.7888}	inf	179.	225.962	{222.295, 230.163}	26.2358	0.101102	newly	120.	75.88	{65., 86.}	36.7667	0.201458	Reff	1.07866	0.650474	{0.645205, 0.655443}	39.6961	ar	3460.	7150.76	{7050., 7222.}	106.669
6	R0	{1.22184, 4.34395}	{1.75613, 15.9244}	{{1.47625, 10.1313}, {2.16167, 20.}}	{43.728, 266.587}	inf	231.	911.834	{578.74, 1361.25}	294.733	0.574662	newly	353.	429.11	{305., 570.}	27.8102	0.103618	Reff	1.01352	1.35557	{1.16634, 1.56806}	33.7497	ar	3524.	7874.89	{6669., 9184.}	123.465
7	R0	{1.23856, 4.59068}	{1.65373, 18.4913}	{{1.46489, 13.5835}, {1.85335, 20.}}	{33.5208, 302.801}	inf	200.	992.326	{709.411, 1322.35}	396.163	0.684822	newly	288.	383.47	{309., 477.}	33.5451	0.120475	Reff	0.970659	1.18156	{1.06672, 1.26334}	21.728	ar	3855.	7769.79	{6867., 8532.}	101.551
8	R0	{1.34531, 10.6689}	{1.55645, 19.7317}	{{1.51712, 19.2102}, {1.59305, 20.}}	{15.6943, 84.9459}	inf	360.	848.356	{781.74, 908.459}	135.655	0.371566	newly	242.	299.1	{271., 327.}	23.595	0.0908624	Reff	1.0562	1.01594	{0.996852, 1.03984}	3.81174	ar	4852.	7302.	{6987., 7563.}	50.4946
9	R0	{1.31775, 5.19226}	{1.67011, 19.0839}	{{1.51124, 14.8625}, {1.83697, 20.}}	{26.7397, 267.544}	inf	400.	1027.27	{808.233, 1247.33}	156.817	0.40356	newly	527.	384.06	{317., 446.}	27.1233	0.141279	Reff	1.00689	1.15339	{1.05364, 1.22561}	14.5909	ar	4708.	7736.49	{6908., 8301.}	64.3265
10	R0	{1.25916, 6.89207}	{1.55464, 19.7553}	{{1.51363, 19.1424}, {1.5943, 20.}}	{23.4665, 186.638}	inf	255.	866.92	{813.407, 919.813}	239.968	0.530375	newly	246.	308.45	{280., 346.}	25.3862	0.0966823	Reff	0.978871	1.05947	{1.02723, 1.08872}	8.35634	ar	3823.	7389.7	{7098., 7640.}	93.2958
11	R0	{1.20377, 5.46566}	{1.61052, 17.8653}	{{1.44186, 13.1535}, {1.81261, 20.}}	{33.7895, 226.865}	inf	165.	803.031	{544.162, 1114.82}	386.686	0.672814	newly	221.	325.97	{234., 406.}	48.2579	0.163327	Reff	0.992749	1.21315	{1.10059, 1.33208}	22.3054	ar	3114.	7209.66	{6422., 8122.}	131.524
12	R0	{1.27878, 2.55804}	{2.30068, 18.5199}	{{1.77164, 10.7365}, {2.71264, 20.}}	{79.9118, 623.986}	inf	209.	2109.01	{1282.32, 2865.01}	909.095	0.990118	newly	522.	834.27	{699., 1024.}	59.8218	0.199872	Reff	0.948983	1.34391	{1.18888, 1.4657}	41.6161	ar	3930.	9849.22	{8633., 10369.}	150.616
13	R0	{1.41227, 5.45696}	{1.58536, 19.7644}	{{1.54322, 19.2833}, {1.63568, 20.}}	{12.256, 262.187}	inf	402.	939.208	{812.96, 1051.44}	133.634	0.366532	newly	505.	330.74	{290., 383.}	34.5069	0.186337	Reff	1.01556	1.00451	{0.968093, 1.03742}	2.08508	ar	4822.	7465.65	{7196., 7716.}	54.8248
14	R0	{1.32917, 7.27593}	{1.57562, 19.6611}	{{1.50874, 18.9929}, {1.63173, 20.}}	{18.5417, 170.221}	inf	374.	939.445	{832.289, 1046.4}	151.189	0.398531	newly	351.	336.73	{300., 380.}	8.50997	0.0382983	Reff	0.994485	1.05515	{1.02242, 1.08579}	6.29482	ar	4758.	7559.21	{7220., 7800.}	58.8737
15	R0	{1.31629, 9.97481}	{1.56228, 19.6538}	{{1.51381, 18.9865}, {1.59615, 20.}}	{18.688, 97.0346}	inf	325.	805.245	{705.484, 922.738}	147.768	0.391937	newly	238.	292.82	{258., 339.}	23.1513	0.0881797	Reff	1.102	1.15498	{1.12715, 1.18001}	4.97936	ar	4543.	7372.26	{7050., 7702.}	62.2773
16	R0	{1.36735, 5.74166}	{1.73307, 18.6969}	{{1.50789, 13.7127}, {1.90687, 20.}}	{26.7467, 225.636}	inf	345.	1080.02	{720.636, 1377.59}	213.05	0.482567	newly	435.	410.23	{308., 501.}	14.2322	0.0663391	Reff	1.05464	1.19498	{1.07766, 1.27767}	13.5119	ar	4516.	8071.68	{6945., 8782.}	78.7352
17	R0	{1.27524, 13.2396}	{1.54004, 19.8452}	{{1.51546, 19.3803}, {1.56661, 20.}}	{20.7649, 49.8931}	inf	189.	781.991	{745.84, 825.956}	313.752	0.616434	newly	111.	278.51	{254., 302.}	150.91	0.398553	Reff	1.19643	1.09446	{1.08216, 1.10472}	8.52262	ar	3731.	7235.98	{7019., 7481.}	93.9421
18	R0	{1.21884, 7.71545}	{1.55115, 19.7398}	{{1.51003, 19.2968}, {1.57886, 20.}}	{27.2643, 155.847}	inf	231.	829.342	{753.207, 895.158}	259.023	0.554329	newly	192.	296.53	{269., 324.}	54.4427	0.187569	Reff	1.02078	1.08592	{1.06579, 1.10158}	6.38123	ar	3080.	7418.77	{7174., 7674.}	140.869
19	R0	{1.34879, 6.71464}	{1.70467, 17.4284}	{{1.54802, 13.7112}, {1.92317, 20.}}	{26.3852, 159.559}	inf	378.	1138.57	{849.45, 1498.06}	201.208	0.466512	newly	370.	455.87	{372., 539.}	23.7649	0.0890017	Reff	0.926888	1.06047	{0.953216, 1.15791}	14.4506	ar	4585.	8016.93	{7246., 8940.}	74.8513
20	R0	{1.37284, 5.75036}	{1.66144, 17.1811}	{{1.43672, 12.4134}, {1.83612, 20.}}	{21.0609, 198.783}	inf	291.	962.469	{584.949, 1311.57}	230.745	0.499945	newly	356.	394.25	{308., 472.}	17.323	0.0694158	Reff	1.04981	1.14016	{1.04155, 1.24408}	9.19748	ar	4750.	7412.86	{6250., 8301.}	56.0602
21	R0	{1.29938, 5.36419}	{1.83734, 18.7459}	{{1.61161, 14.5036}, {2.07069, 20.}}	{41.4011, 249.464}	inf	347.	1255.41	{850.008, 1560.25}	261.789	0.549417	newly	456.	478.27	{385., 550.}	12.0197	0.0510511	Reff	0.968298	1.20471	{1.08718, 1.29597}	24.4157	ar	4337.	8498.19	{7587., 9082.}	95.9463
22	R0	{1.31882, 7.47626}	{1.55516, 19.7873}	{{1.49856, 19.3393}, {1.60478, 20.}}	{17.9206, 164.669}	inf	278.	869.493	{776.961, 1004.99}	212.767	0.493074	newly	256.	308.19	{277., 352.}	20.5508	0.0791418	Reff	1.04292	1.0565	{1.01677, 1.08346}	2.56132	ar	3977.	7312.32	{6984., 7640.}	83.8652
23	R0	{1.26215, 3.46375}	{1.8949, 18.8115}	{{1.65959, 14.8147}, {2.15459, 20.}}	{50.1329, 443.097}	inf	212.	1294.64	{956.523, 1554.17}	510.677	0.778699	newly	394.	498.76	{411., 575.}	27.2081	0.101686	Reff	0.984098	1.26322	{1.12921, 1.37749}	28.3632	ar	3607.	8624.83	{7829., 9283.}	139.114
24	R0	{1.16712, 4.19464}	{1.55486, 19.4714}	{{1.49706, 18.6549}, {1.63016, 20.}}	{33.2215, 364.196}	inf	126.	873.483	{774.761, 988.967}	593.24	0.839052	newly	188.	317.59	{275., 353.}	68.9309	0.225911	Reff	0.946418	1.10502	{1.03542, 1.14624}	16.7584	ar	3000.	7521.22	{7123., 7862.}	150.707
25	R0	{1.22751, 3.34517}	{1.76667, 17.2223}	{{1.47928, 12.9577}, {2.08152, 20.}}	{43.9232, 414.842}	inf	170.	831.27	{508.928, 1160.59}	388.983	0.67068	newly	366.	362.73	{283., 472.}	15.9208	0.0706342	Reff	1.07714	1.40839	{1.23706, 1.58117}	30.7592	ar	3086.	7806.15	{6565., 8819.}	152.954
26	R0	{1.18747, 5.88083}	{1.54398, 19.8463}	{{1.50218, 19.4546}, {1.58892, 20.}}	{30.0225, 237.474}	inf	219.	910.718	{840.69, 989.671}	315.853	0.618131	newly	259.	320.72	{289., 354.}	23.8378	0.0915267	Reff	0.891909	1.01997	{0.99277, 1.04566}	14.3577	ar	3614.	7435.69	{7142., 7685.}	105.747
27	R0	{1.29023, 4.76202}	{1.64546, 19.4703}	{{1.52665, 18.8243}, {1.73785, 20.}}	{27.5323, 308.866}	inf	314.	1066.61	{814.305, 1240.31}	239.686	0.525792	newly	412.	383.26	{305., 443.}	10.8252	0.0515014	Reff	0.948061	1.06095	{1.00783, 1.1124}	11.9295	ar	4245.	7696.69	{7109., 8124.}	81.3119
28	R0	{1.2258, 12.9056}	{1.51513, 19.998}	{{1.50793, 20.}, {1.51665, 20.}}	{23.6037, 54.9564}	inf	204.	140.565	{136.563, 141.797}	31.0955	0.162305	newly	111.	45.61	{37., 52.}	58.9099	0.389987	Reff	0.977943	0.621584	{0.62005, 0.624377}	36.4397	ar	3639.	7741.66	{7665., 7819.}	112.741
29	R0	{1.34702, 6.29049}	{1.62984, 18.4697}	{{1.47445, 13.8176}, {1.75132, 20.}}	{20.9958, 193.613}	inf	319.	989.055	{739.463, 1249.71}	210.049	0.483058	newly	360.	375.16	{312., 451.}	11.6722	0.0486467	Reff	0.99693	1.08212	{0.978393, 1.16164}	9.605	ar	4560.	7626.81	{6798., 8288.}	67.2546
30	R0	{1.37931, 6.78741}	{1.64507, 19.0632}	{{1.48053, 14.5882}, {1.82329, 20.}}	{19.2675, 180.862}	inf	364.	1028.23	{769.683, 1246.87}	182.481	0.442697	newly	361.	379.33	{300., 451.}	14.5235	0.0609054	Reff	0.975724	1.07141	{0.982215, 1.13105}	10.2551	ar	4667.	7782.3	{6936., 8419.}	66.7517
31	R0	{1.16062, 4.47529}	{1.77585, 17.6807}	{{1.50013, 13.3857}, {2.08591, 20.}}	{53.0089, 295.075}	inf	237.	898.321	{601.413, 1220.19}	279.038	0.563019	newly	360.	378.27	{275., 474.}	18.6639	0.0780237	Reff	0.985599	1.39124	{1.22639, 1.55655}	41.1565	ar	3037.	7919.82	{6860., 8868.}	160.778
32	R0	{1.37647, 18.7122}	{1.52906, 19.9483}	{{1.49446, 19.7232}, {1.55959, 20.}}	{11.0852, 6.6061}	inf	357.	211.852	{196.972, 230.666}	40.6578	0.227612	newly	145.	69.14	{58., 79.}	52.3172	0.324994	Reff	1.02478	0.623014	{0.614522, 0.631254}	39.2052	ar	4824.	7553.35	{7375., 7706.}	56.5786
33	R0	{1.35084, 6.08841}	{1.75814, 19.5154}	{{1.61718, 19.1962}, {1.89773, 20.}}	{30.1515, 220.534}	inf	485.	1273.86	{1027.46, 1538.48}	162.652	0.414516	newly	533.	462.22	{388., 547.}	14.7955	0.0717708	Reff	0.972335	1.14041	{1.0514, 1.19945}	17.2856	ar	4759.	8467.66	{7956., 8965.}	77.9294
34	R0	{1.36912, 6.316}	{1.59648, 19.489}	{{1.51073, 18.7496}, {1.65891, 20.}}	{16.606, 208.566}	inf	294.	917.293	{763.489, 1084.16}	212.005	0.490167	newly	335.	335.69	{283., 389.}	10.4388	0.0453558	Reff	1.06983	1.12312	{1.07332, 1.17781}	5.60286	ar	4537.	7421.3	{6996., 7850.}	63.5728
35	R0	{1.35831, 7.09038}	{1.74861, 18.9419}	{{1.5353, 14.3079}, {1.90742, 20.}}	{28.7343, 167.15}	inf	375.	1217.41	{890.174, 1472.37}	224.642	0.500847	newly	358.	445.08	{347., 514.}	25.8939	0.0968682	Reff	0.949459	1.05184	{0.990284, 1.13399}	10.7834	ar	4667.	8080.4	{7118., 8679.}	73.1391
36	R0	{1.28315, 5.89938}	{1.87796, 18.7773}	{{1.58748, 14.3754}, {2.11893, 20.}}	{46.3554, 218.293}	inf	365.	1368.61	{907.535, 1683.33}	274.962	0.56455	newly	398.	516.62	{419., 598.}	30.2613	0.11173	Reff	0.92297	1.16964	{1.06827, 1.2463}	26.7253	ar	4460.	8616.47	{7474., 9274.}	93.1944
37	R0	{1.19303, 2.75113}	{1.81141, 17.1625}	{{1.49207, 12.2817}, {2.11238, 20.}}	{51.8324, 523.835}	inf	171.	1066.56	{652.311, 1407.22}	523.717	0.776297	newly	357.	454.77	{352., 553.}	29.986	0.111008	Reff	0.971365	1.32957	{1.15243, 1.48179}	36.8767	ar	3142.	8123.38	{6775., 9041.}	158.542
38	R0	{1.36033, 7.8088}	{1.66918, 19.1575}	{{1.52667, 17.4532}, {1.87229, 20.}}	{22.7039, 145.332}	inf	377.	1038.68	{797.871, 1298.06}	175.513	0.433402	newly	348.	384.62	{317., 460.}	15.9425	0.0637023	Reff	1.02419	1.14923	{1.05453, 1.21735}	12.3488	ar	4941.	7810.13	{7145., 8485.}	58.0678
39	R0	{1.27353, 3.70845}	{1.90676, 19.1344}	{{1.74062, 15.2095}, {2.12522, 20.}}	{49.7221, 415.966}	inf	255.	1373.37	{1128.81, 1596.3}	438.576	0.725933	newly	460.	514.63	{448., 570.}	13.5543	0.0544316	Reff	0.944322	1.21955	{1.13304, 1.30273}	29.1457	ar	3725.	8740.09	{8224., 9280.}	134.633
40	R0	{1.19983, 12.1486}	{1.48908, 19.6116}	{{1.47083, 19.5111}, {1.55578, 19.7867}}	{24.1074, 61.4306}	inf	151.	116.359	{114.182, 118.326}	22.9407	0.113235	newly	79.	38.35	{32., 46.}	51.4557	0.318446	Reff	0.976302	0.617709	{0.600478, 0.622833}	36.7297	ar	3619.	7300.43	{7172., 7528.}	101.725
41	R0	{1.20935, 2.67169}	{2.26475, 17.9917}	{{1.69216, 10.5216}, {2.53881, 20.}}	{87.2704, 573.42}	inf	236.	1808.37	{1055.44, 2226.73}	666.256	0.865671	newly	573.	757.17	{623., 871.}	32.4241	0.118035	Reff	0.945228	1.49989	{1.24057, 1.66493}	58.6807	ar	3429.	9677.63	{8259., 10247.}	182.229
42	R0	{1.23313, 4.74781}	{1.53306, 19.7634}	{{1.46867, 19.2824}, {1.59736, 20.}}	{24.323, 316.263}	inf	192.	836.213	{755.735, 931.444}	335.528	0.637315	newly	278.	299.71	{264., 333.}	10.1115	0.0413517	Reff	0.975036	1.07922	{1.04062, 1.1204}	10.7277	ar	3835.	7419.63	{7107., 7743.}	93.4714
43	R0	{1.26105, 2.39713}	{2.06791, 18.4615}	{{1.75196, 16.3169}, {2.29112, 20.}}	{63.9829, 670.15}	inf	151.	1690.05	{1170.02, 2146.08}	1019.24	1.03878	newly	396.	662.65	{525., 797.}	67.3359	0.218923	Reff	0.95865	1.26107	{1.15158, 1.36872}	31.5461	ar	3723.	9257.17	{8430., 9716.}	148.648
44	R0	{1.36948, 8.88006}	{1.58461, 19.6963}	{{1.52567, 18.8735}, {1.63868, 20.}}	{15.709, 121.804}	inf	376.	885.092	{771.289, 997.948}	135.397	0.369511	newly	309.	320.29	{278., 357.}	8.46278	0.0355647	Reff	1.06984	1.127	{1.0859, 1.17765}	5.62053	ar	4437.	7348.51	{6977., 7673.}	65.6189
45	R0	{1.34882, 3.3791}	{2.50027, 18.3515}	{{2.18946, 14.8893}, {2.73208, 20.}}	{85.3672, 443.088}	inf	365.	2062.36	{1707.63, 2397.39}	465.03	0.743836	newly	717.	857.53	{744., 946.}	21.3598	0.0836373	Reff	1.04641	1.56165	{1.3777, 1.68938}	49.2379	ar	4422.	9699.54	{9266., 10043.}	119.347
46	R0	{1.23774, 4.34917}	{1.71731, 17.2717}	{{1.44166, 12.9757}, {2.03544, 20.}}	{38.7456, 297.126}	inf	261.	933.656	{581.909, 1191.59}	257.723	0.536168	newly	370.	391.28	{287., 464.}	16.1622	0.0682537	Reff	0.996752	1.26196	{1.11987, 1.41533}	26.6076	ar	3685.	7657.11	{6389., 8675.}	107.791
47	R0	{1.34327, 4.78811}	{1.87841, 18.5537}	{{1.50773, 13.3551}, {2.0926, 20.}}	{39.8388, 287.494}	inf	281.	1125.05	{638.577, 1403.39}	300.374	0.588514	newly	448.	441.32	{345., 530.}	13.6964	0.0614812	Reff	1.0953	1.31119	{1.16589, 1.38269}	19.7105	ar	4517.	8174.16	{6698., 8850.}	80.9644
48	R0	{1.24366, 4.25814}	{1.68774, 19.25}	{{1.5077, 18.3161}, {1.84422, 20.}}	{35.7078, 352.076}	inf	209.	1069.01	{752.436, 1350.42}	411.487	0.699738	newly	310.	397.04	{318., 487.}	29.2129	0.106806	Reff	0.987217	1.16276	{1.08791, 1.2142}	17.7851	ar	3678.	7823.33	{7068., 8426.}	112.706
49	R0	{1.36017, 7.5415}	{1.52419, 19.5525}	{{1.48666, 19.0101}, {1.56262, 20.}}	{12.0586, 159.266}	inf	190.	869.732	{828.093, 919.871}	357.754	0.660113	newly	183.	310.9	{289., 335.}	69.8907	0.229424	Reff	1.12513	1.03762	{1.02021, 1.06363}	7.77803	ar	4178.	7486.05	{7240., 7735.}	79.1778
50	R0	{1.25333, 6.63578}	{1.55766, 19.6439}	{{1.4913, 19.386}, {1.6258, 20.}}	{24.2814, 196.03}	inf	193.	910.921	{839.271, 1005.12}	371.98	0.672856	newly	204.	325.42	{296., 350.}	59.5196	0.201681	Reff	0.945763	1.03415	{0.991306, 1.06564}	9.40404	ar	3401.	7779.17	{7405., 8043.}	128.732
